# Immune surveillance as a pharmacological target in the early stages of cancer

**DOI:** 10.3389/fmolb.2025.1643024

**Published:** 2025-07-25

**Authors:** Hongfei Jiang, Zhun Wei, Haibo Zhao

**Affiliations:** ^1^ The Affiliated Hospital of Qingdao University and Qingdao Cancer Institute, Qingdao, China; ^2^ Department of Pharmacology, School of Pharmacy, Qingdao University Medical College, Qingdao University, Qingdao, China

**Keywords:** immune surveillance, tumor microenvironment (TME), early cancer interception, immunoprevention, pharmacological modulation

## 1 Introduction

Immune surveillance is a critical biological mechanism by which the body’s immune system identifies and eliminates early-stage tumor cells before they progress into clinically detectable malignancies (Catena et al., 2025; Zhang Y. et al., 2024). The concept of immune surveillance, first proposed decades ago, has significantly reshaped our understanding of cancer biology, emphasizing the intricate interactions between tumor initiation and host immunity (Burnet, 1970; Dunn et al., 2004). Central to this protective mechanism are diverse cellular players, including natural killer (NK) cells, cytotoxic T lymphocytes (CTLs), dendritic cells (DCs), and various cytokines, all collaborating within a highly coordinated network to prevent tumor establishment and growth (Coënon et al., 2024). While traditional immunotherapies target advanced stages, emerging evidence supports targeting immune surveillance specifically during precancerous stages, Recent studies have demonstrated that CD8^+^ T cells actively infiltrate early lesions and exert tumor-suppressive effects in melanoma models (Eyles et al., 2010). This suggests that immune surveillance remains active and targetable at precancerous stages.

Although substantial progress has been achieved in harnessing the immune system to treat advanced cancers, particularly through immune checkpoint inhibitors and adoptive cell therapies, these strategies often face challenges, such as limited efficacy, immune-related adverse events, and resistance mechanisms emerging in later stages (Rui et al., 2023). Consequently, shifting the focus to the early stages of cancer progression or even precancerous conditions could significantly enhance therapeutic outcomes (Faupel-Badger et al., 2024). Targeting immune surveillance pharmacologically at these initial stages provides an appealing strategy, potentially halting malignant transformation long before invasive disease becomes established (Liu et al., 2025). In this article, we use the term “precancerous lesions” to refer to histologically abnormal but non-invasive tissue changes (e.g., ductal carcinoma *in situ*), while “early-stage cancer” denotes lesions with minimal invasion but limited metastatic potential. Although distinct, both represent windows where immune surveillance may be effectively modulated. We further align this usage with the immunoediting framework, in which “early-stage” corresponds to the “elimination” and “equilibrium” phases—where immune control is still partially intact. This classification is supported by recent literature advocating a multi-dimensional definition of precancer and early transformation, integrating histological, molecular, and immune parameters (Faupel-Badger et al., 2024).

Nevertheless, despite the potential benefits, our current understanding of immune surveillance at early or precancerous stages remains incomplete (Zhou R. et al., 2024). Several key gaps persist, including how early tumors initially evade or disrupt immune recognition, the precise role of the tumor microenvironment in suppressing early immune responses, and how immune-editing shapes tumor evolution from precancer to cancer. Additionally, existing therapeutic strategies developed for advanced tumors are generally unsuitable or untested for early-stage intervention, due to uncertainties regarding risk-benefit ratios and the difficulty of identifying appropriate patient populations for such interventions (Crosby et al., 2022).

In this opinion article, we explore the potential of immune surveillance as a pharmacological target at the earliest stages of cancer. By discussing current limitations, emerging therapeutic strategies, and opportunities presented by advanced analytical tools, such as single-cell multi-omics and artificial intelligence, we aim to highlight innovative pathways for early immune-based interventions. Importantly, we also emphasize the dynamic role of the tumor microenvironment (TME)—a complex ecosystem comprising immunoregulatory cells such as regulatory T cells (Tregs), myeloid-derived suppressor cells (MDSCs), and tumor-associated macrophages (TAMs)—in shaping the efficacy of immune surveillance during precancerous transitions. Understanding and pharmacologically manipulating these early immunosuppressive signals within the TME could provide new leverage points for intercepting malignant progression. Ultimately, prioritizing pharmacological modulation of immune surveillance at the onset of cancer development may significantly improve patient prognosis, reduce cancer-related mortality, and open new frontiers in precision oncology.

In this Opinion article, we propose that the earliest stages of immune surveillance breakdown—particularly involving spatially localized immune checkpoint upregulation and metabolic suppression—represent a therapeutically actionable window (Gajewski et al., 2013). We hypothesize that immune recalibration, rather than broad immune activation, offers a safer and more durable approach for intercepting malignancy at the precancerous phase. This perspective challenges conventional strategies that prioritize maximal immune stimulation, and instead advocates for minimal, precision-guided interventions to restore immune equilibrium in high-risk tissues.

## 2 Immune surveillance and TME crosstalk in early tumor initiation

Building upon the foundational concept introduced above, immune surveillance engages both innate and adaptive immune arms to eliminate early transformed cells in a dynamic and tissue-specific manner. Immune surveillance represents a crucial first line of defense against tumor initiation and involves coordinated efforts from both innate and adaptive immune cells (Dunn et al., 2002). The innate immune system, characterized by rapid and non-specific responses, includes NK cells, DCs, and macrophages. NK cells play a crucial role in the elimination phase of cancer immunoediting by recognizing and lysing transformed cells that downregulate MHC class I molecules or express stress ligands. Their activity is particularly important in the earliest stages of tumor formation, where adaptive immunity has not yet been fully engaged. Similarly, macrophages—especially in their M1-polarized state—contribute to early antitumor responses through phagocytosis, secretion of pro-inflammatory cytokines (e.g., IL-12, TNF-α), and support of T cell recruitment. However, during tumor progression, macrophages often undergo phenotypic reprogramming toward the M2-like, tumor-promoting phenotype. This early plasticity makes them a potential pharmacological target to maintain effective innate immune surveillance (Coënon et al., 2024; Zhou L. et al., 2024). NK cells can recognize and eliminate cells displaying altered expression of stress-induced ligands or reduced expression of major histocompatibility complex (MHC) class I molecules, typical of transformed cells (Zhang H. et al., 2024). Dendritic cells are essential for bridging innate and adaptive immunity, efficiently capturing and processing tumor-derived antigens to prime tumor-specific T cells (Gardner et al., 2020). Macrophages, which possess dual functional states (M1 pro-inflammatory and M2 anti-inflammatory phenotypes), can initially exert tumoricidal activities but may be co-opted by the evolving TME to support tumor progression (Zhou L. et al., 2024).

The adaptive immune system, characterized by specificity and immunological memory, plays a central role in immune surveillance through CD8^+^ cytotoxic T lymphocytes (CTLs) and CD4^+^ helper T cells (Afzali and Korn, 2025; Jiang et al., 2023). CTLs mediate direct killing of tumor cells via recognition of tumor-specific neoantigens presented by MHC class I molecules. CD4^+^ helper T cells orchestrate antitumor immunity by enhancing CTL activity, modulating macrophage functions, and promoting B cell-mediated antibody responses. Tregs, although critical in maintaining immune homeostasis, may facilitate tumor immune evasion by suppressing antitumor immune responses when disproportionately expanded in the tumor microenvironment. At the molecular level, critical pathways such as PD-1/PD-L1, CTLA-4, and TIM-3 signaling are implicated early in immune surveillance disruption. Specifically, upregulation of immune checkpoints on precancerous cells can induce early T-cell exhaustion, facilitating immune evasion. Furthermore, cytokines such as TGF-β, IL-10, and prostaglandin E2 (PGE2) secreted by early neoplastic cells and immunosuppressive TME cells (e.g., MDSCs, TAMs) critically alter molecular signaling cascades, inhibiting effective immune responses at early stages.

Recent studies indicate that PD-L1 expression can emerge surprisingly early during the transition from normal epithelium to dysplasia, often driven by interferon-γ (IFN-γ) secreted by tumor-infiltrating lymphocytes (TILs). This early upregulation may serve as a compensatory feedback mechanism but inadvertently facilitates T cell exhaustion (Zebley et al., 2024). Similarly, TGF-β produced by pre-malignant epithelial cells and stromal fibroblasts not only dampens cytotoxic T cell activity but also promotes the expansion of regulatory T cells (Tregs) and cancer-associated fibroblasts (CAFs), reinforcing early immunosuppression *in situ* (Lakins et al., 2018). These data support the notion that immune checkpoint signaling and suppressive cytokine cascades are not exclusive to late-stage tumors but initiate during early immune editing phases, making them viable early pharmacological targets. Furthermore, spatial transcriptomic analyses have revealed that early lesions often exhibit regional heterogeneity in immune checkpoint molecule expression, suggesting that localized immunosuppressive niches may drive clonal escape and immune evasion (Takano et al., 2024).

Despite robust immune surveillance mechanisms, transformed cells can evade immune detection through the dynamic process termed immunoediting, encompassing three phases: elimination, equilibrium, and escape (Mittal et al., 2014). Initially, immune cells successfully eliminate nascent tumor cells. However, some tumor variants survive and enter a prolonged equilibrium state, maintained by balanced interactions between immune cells and tumor cells. Over time, selective pressure imposed by immune cells can drive the emergence of tumor cell clones capable of escaping immune recognition, often by altering antigen presentation, secreting immunosuppressive cytokines, and recruiting immunoregulatory cells into the TME. Importantly, T cell dysfunction begins early in this process. During the equilibrium phase, persistent antigen exposure leads to progressive CD8^+^ T cell exhaustion, characterized by upregulation of inhibitory receptors such as PD-1, TIM-3, and LAG-3, along with diminished cytokine production and cytolytic capacity (Chi et al., 2023). Concurrently, dendritic cells may exhibit reduced co-stimulatory molecule expression and impaired cross-presentation, weakening T cell priming. MDSCs further amplify immune suppression by producing arginase and reactive oxygen species, thereby metabolically paralyzing T cells and inhibiting antigen presentation. This multilayered suppression results in failure of tumor clearance and supports immune escape (Chen et al., 2023).

Clinical and preclinical studies have underscored the significance of intact immune surveillance (Sauna et al., 2023). For instance, immunodeficient mouse models exhibit significantly elevated spontaneous and carcinogen-induced tumor incidence, clearly demonstrating immune surveillance’s role in limiting tumorigenesis (Shankaran et al., 2001). Additionally, human epidemiological studies highlight increased cancer risk among immunocompromised individuals, reinforcing the clinical relevance of immune surveillance (Grulich et al., 2007; Vajdic and van Leeuwen, 2009). Consistently, preclinical studies have shown that mice deficient in IFN-γ or lymphocytes exhibit over a 60% increase in spontaneous tumor formation, confirming the essential role of immune surveillance during early tumorigenesis (Shankaran et al., 2001). Together, these findings emphasize immune surveillance as a powerful yet imperfect barrier to cancer initiation, thus presenting pharmacological enhancement of this innate defense as an attractive therapeutic goal for intercepting cancer at its earliest stages.

## 3 Pharmacological challenges in targeting immune surveillance within the TME

The advent of cancer immunotherapy, particularly immune checkpoint inhibitors and adoptive T-cell therapies, has revolutionized treatment paradigms for advanced cancers. These therapies primarily work by reactivating suppressed immune responses or directly enhancing the tumor-specific cytotoxic functions of immune cells (Topalian Suzanne et al., 2015). Despite their transformative impact in oncology, current immunotherapeutic approaches have critical limitations when considered for targeting immune surveillance in precancerous or early-stage cancers.

First, current immunotherapies were originally developed and clinically validated in the context of advanced-stage malignancies, characterized by significant tumor burdens and extensive immune suppression. Therapies such as PD-1/PD-L1 inhibitors (e.g., nivolumab, pembrolizumab) or CTLA-4 inhibitors (e.g., ipilimumab) act by overcoming profound immune checkpoint-mediated inhibition that is typically present in established cancers (Buchbinder and Desai, 2016). However, early-stage lesions often do not exhibit well-established checkpoint-mediated immune suppression, making it unclear whether such potent immune modulators would be effective or safe if administered at this stage. The risk of overstimulation and immune-related adverse events (irAEs), such as autoimmunity or tissue inflammation, also remains a significant concern when considering treatment for lesions that may spontaneously regress or never progress (Ramos-Casals et al., 2020; Morgado et al., 2020).

Second, early detection and intervention rely heavily on identifying biomarkers that predict malignant transformation, yet clinically validated biomarkers to stratify early-stage lesions according to progression risk remain lacking. Without precise markers, applying powerful immune-modulating drugs could inadvertently result in overtreatment of lesions with a low likelihood of progression, leading to unnecessary patient anxiety, health risks, and increased healthcare costs (Lin et al., 2023).

Third, tumor heterogeneity at early stages adds another layer of complexity. Precancerous or early-stage lesions may differ significantly from advanced tumors in terms of neoantigen presentation, immune cell infiltration, and microenvironmental composition (Jia et al., 2022). Thus, therapies effective against established tumors may fail to activate sufficient or specific immune responses required to eliminate or control precancerous cells. Additionally, emerging evidence suggests that the immune landscape evolves dramatically during early cancer development, necessitating a deeper understanding of immune dynamics at these initial phases.

Finally, current immunotherapeutics have limited efficacy in cancers with inherently low immunogenicity (often termed “cold tumors”). Early-stage lesions frequently possess limited mutations and express fewer neoantigens, potentially reducing their immunogenicity. Therefore, treatments designed to bolster adaptive immune responses against highly mutated, advanced cancers might be ineffective against early lesions unless combined with strategies to enhance neoantigen visibility or antigen presentation (Wang et al., 2020; Xie et al., 2023).

In summary, while contemporary immunotherapies represent remarkable advancements for advanced cancers, critical limitations, including uncertain risk-benefit profiles, the lack of early-stage biomarkers, insufficient knowledge of early immune dynamics, and low immunogenicity of early lesions, currently constrain their adaptation to pharmacologically targeting immune surveillance in precancerous and early-stage disease. Moreover, some experts argue that intervening too early with immune-modulating agents may risk overtreatment, autoimmunity, or disruption of immune homeostasis, especially in lesions with uncertain malignant potential. Addressing these limitations will be essential to fully exploit immune surveillance as a preventive pharmacological strategy against cancer.

## 4 Emerging pharmacological strategies to enhance immune surveillance at early cancer stages

Accumulating experimental evidence supports the potential efficacy of immune interventions at the early stages of tumor formation. Specifically, studies in melanoma provide robust examples where immune surveillance mechanisms effectively control or eliminate early-stage lesions (Eyles et al., 2010). For instance, preclinical mouse models have demonstrated that antigen-specific CD8^+^ T cells infiltrating early melanoma lesions can effectively mediate complete tumor regression or sustained growth control (Matsushita et al., 2012). In particular, early-stage melanomas express high levels of immunogenic neoantigens, providing ideal targets for immunotherapeutic interventions (Anagnostou et al., 2017). Additionally, clinical observations in patients with early melanoma lesions treated with immunotherapies such as checkpoint inhibitors or neoantigen vaccines have reported significant immune responses and improved lesion control compared to advanced-stage tumors (Ott et al., 2017). These findings strongly advocate that the window for effective immune-based intervention exists primarily at early tumor stages, when the tumor burden and immune suppression are minimal, and immune cells retain functional responsiveness.

**FIGURE 1:**
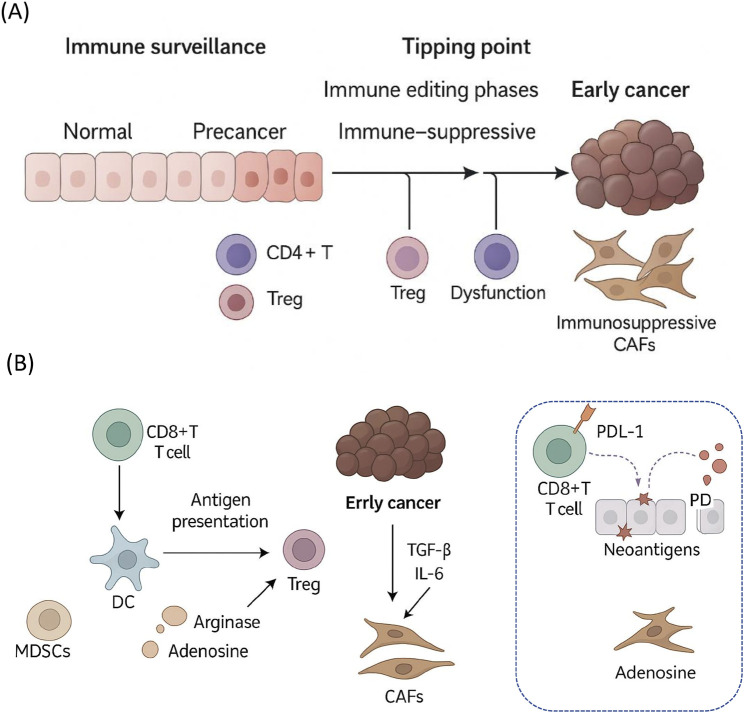
Immune surveillance failure and early immunosuppressive remodeling during the precancer-to-cancer transition. This schematic illustrates the temporal and cellular progression from effective immune surveillance to immune escape. **(A)** Overview of the immune surveillance phases during early tumorigenesis. In normal tissues, immune homeostasis is maintained. During precancerous stages, immune editing begins with infiltration of CD4^+^ T cells and Tregs. At the tipping point, immunosuppressive mechanisms intensify, including T cell dysfunction and the emergence of immunosuppressive CAFs, ultimately facilitating early cancer formation. **(B)** Key mechanisms of early immune evasion. Myeloid-derived suppressor cells (MDSCs) and tumor-derived factors (e.g., arginase, adenosine) inhibit antigen presentation by dendritic cells (DCs) and promote Treg differentiation. Early tumors and CAFs secrete TGF-β and IL-6 to reinforce immune suppression. Neoantigen-specific CD8^+^ T cells become functionally impaired through PD-1/PD-L1 interactions, leading to immune escape and tumor progression.

The pharmacological enhancement of immune surveillance at early cancer stages represents an exciting and rapidly evolving frontier in cancer prevention and therapy. Recent advances in immunology, molecular biology, and pharmacology have yielded innovative approaches to address current limitations and specifically target precancerous or early neoplastic lesions. Several emerging strategies stand out, offering promise for future clinical translation (Roeser et al., 2015). To better contextualize the dynamic interplay between immune surveillance and early tumor evolution, Figure 1 provides a conceptual overview of cellular and molecular events during the precancer-to-cancer transition. It illustrates key immune cell subsets (e.g., CD8^+^ T cells, Tregs, MDSCs), immunosuppressive cytokines (TGF-β, IL-6), and inhibitory pathways such as PD-1/PD-L1 signaling. These events are shown progressing over time, reflecting opportunities for pharmacological interventions such as checkpoint blockade, Treg modulation, and dendritic cell activation to restore immune equilibrium before full tumor establishment. As shown, during the precancerous phase, immune surveillance mechanisms—primarily involving CD8^+^ cytotoxic T cells and CD4^+^ helper T cells—actively monitor and attempt to eliminate aberrant cells. However, a gradual shift occurs as immune evasion mechanisms intensify, including T cell dysfunction, expansion of immunosuppressive Tregs, and the emergence of CAFs. These changes create an immune-suppressive microenvironment, facilitating malignant progression. This timeline underscores critical intervention points where pharmacological strategies could be leveraged to restore immune vigilance and prevent cancer development.

### 4.1 Novel cancer vaccines targeting precancer-specific neoantigens

One significant advance involves personalized vaccines designed to elicit potent immune responses against tumor-specific or precancer-specific neoantigens. Neoantigens, derived from somatic mutations unique to early transformed cells, present ideal targets for selective immune recognition (Yarchoan et al., 2024). Recent improvements in sequencing technology, bioinformatics, and peptide synthesis have facilitated the development of personalized neoantigen-based vaccines. Early preclinical studies demonstrate that vaccination with neoantigens derived from early-stage lesions induces robust cytotoxic T-cell responses, leading to effective control or even regression of preclinical tumor models (Braun et al., 2025). Additionally, emerging data from early-phase clinical trials suggest that personalized neoantigen vaccines are safe and immunologically effective in stimulating targeted immune responses, especially when applied in the adjuvant or neoadjuvant setting (Keskin et al., 2019). These vaccines work by presenting tumor-specific mutated peptides to dendritic cells, thereby priming CD8^+^ cytotoxic T cells and establishing durable immune memory against early neoplastic clones. For instance, in a phase I study involving melanoma patients, personalized neoantigen vaccines elicited strong CD4^+^ and CD8^+^ T cell responses in all participants, with 60% remaining recurrence-free at a median follow-up of 25 months (Ott et al., 2017).

### 4.2 Modulation of innate immunity through pattern recognition receptors

Another promising strategy focuses on enhancing innate immune responses using synthetic agonists of pattern recognition receptors (PRRs), a class of innate sensors that detect pathogen- or danger-associated signals, particularly Toll-like receptors (TLRs) and stimulator of interferon genes (STING) pathways. Pharmacological stimulation of these innate immune sensors can trigger potent inflammatory responses and subsequently prime adaptive immunity against early transformed cells. For example, TLR agonists such as imiquimod (TLR7 agonist) have demonstrated efficacy in treating precancerous lesions, such as cervical intraepithelial neoplasia, by promoting local immune activation and facilitating regression (Inayama et al., 2023). A recent meta-analysis reported that topical imiquimod achieved complete histological regression in up to 73% of patients with cervical or vaginal intraepithelial neoplasia (Inayama et al., 2023). Similarly, STING agonists induce type I interferon production, driving robust immune activation in the tumor microenvironment (Wang et al., 2024). Mechanistically, PRR agonists activate downstream signaling cascades such as NF-κB and IRF3, leading to type I IFN production and enhanced antigen presentation. Preclinical studies suggest that PRR agonists combined with traditional preventive measures or local immunotherapies might greatly enhance early immune-mediated tumor clearance (Shekarian et al., 2017).

### 4.3 Reprogramming the precancer microenvironment

The precancer microenvironment is increasingly recognized as a critical determinant in early cancer progression. A pharmacological approach gaining traction is the reprogramming of immunosuppressive components in the early tumor microenvironment, such as Tregs, TAMs, and MDSCs. Agents targeting metabolic pathways involved in immunosuppressive cell differentiation, including inhibitors of IDO (indoleamine 2,3-dioxygenase), arginase, or adenosine signaling pathways, are undergoing active investigation (Komiya and Huang, 2018; Prendergast et al., 2018). These drugs aim to shift the microenvironment toward a pro-inflammatory phenotype, facilitating enhanced immune surveillance and tumor cell clearance. These metabolic inhibitors reduce local immunosuppression by restoring L-arginine availability and reversing T cell anergy and Treg polarization within the tumor microenvironment.

### 4.4 Synthetic biomarkers and liquid biopsy approaches

The development of synthetic biomarkers and advanced liquid biopsy techniques provides powerful new tools for real-time monitoring of immune activity and early tumor progression. Synthetic biomarkers, designed to be cleaved or activated by tumor-specific enzymes, can amplify minute biological signals from early-stage tumors, significantly improving detection sensitivity (Hao et al., 2023). Moreover, liquid biopsy technologies, including circulating tumor DNA (ctDNA), circulating immune cell signatures, exosomes, and tumor-derived microRNAs, facilitate non-invasive monitoring of immunological changes associated with early tumorigenesis, allowing for timely therapeutic intervention (Ma et al., 2024).

### 4.5 Integration of single-cell multi-omics and artificial intelligence (AI)

Advanced single-cell multi-omics technologies combined with AI-driven analysis offer a revolutionary approach to dissecting early cancer immune dynamics and uncovering actionable pharmacological targets. Single-cell RNA sequencing, spatial transcriptomics, and proteomics have provided unprecedented insights into immune cell heterogeneity and interactions within early cancer lesions. When integrated with machine learning algorithms, these tools can identify novel immune-related biomarkers, predict lesion progression, and guide personalized pharmacological intervention strategies. This approach allows precise targeting of immune modulatory treatments to those individuals most likely to benefit, thereby avoiding overtreatment and improving outcomes (Sabit et al., 2025; Li et al., 2024; Cai et al., 2022).

### 4.6 Mechanism-guided rationale for early intervention

Mechanistically, early-stage immune escape often initiates with localized checkpoint signaling and suppressive cytokines rather than global immunoparalysis seen in advanced tumors. For instance, early PD-L1 upregulation on epithelial or stromal cells may be driven by IFN-γ released from TILs, suggesting an ongoing, though faltering, immune response. This provides a rationale for intervening before T cells become terminally exhausted. At this stage, checkpoint blockade may restore effector functions more effectively than in late-stage tumors with irreversible exhaustion signatures (Zebley et al., 2024).

Recent advancements in single-cell multi-omics have significantly enhanced our understanding of molecular heterogeneity and dynamic immune interactions at precancerous stages. For example, single-cell RNA sequencing combined with spatial transcriptomics has enabled precise identification of unique molecular signatures associated with early-stage immune evasion. Integration with AI-based predictive analytics further allows high-resolution characterization of cell-cell communication networks, revealing novel targets such as immune checkpoint receptors or cytokine-driven signaling pathways uniquely upregulated during early tumorigenesis. A comparative summary of emerging pharmacological strategies—including their immune targets, mechanisms, clinical status, and translational considerations—is provided in Table 1. This framework facilitates a better understanding of where each intervention fits within the early cancer interception paradigm.

**TABLE 1:** Summary of emerging pharmacological strategies for enhancing early immune surveillance.

Strategy/Agent	Target (pathway/cell)	Mechanism of action	Clinical stage	Advantages and limitations
Neoantigen-based vaccines	Dendritic cells/CD8^+^ T cells	Induce antigen-specific T cell response via personalized mutated peptides	Phase I/II	Highly specific and immunogenic; complex design and delivery logistics
STING agonists	PRRs/innate immune cells	Activate type I IFN response and antigen presentation	Preclinical/Phase I	Potent innate activation; risk of inflammation and off-target effects
IDO inhibitors (e.g., Epacadostat)	Tregs/MDSCs	Block tryptophan catabolism to reverse immune suppression	Phase I/II	Reprograms TME; limited efficacy as monotherapy in late-stage trials
Arginase inhibitors	TME metabolic pathways/T cells	Restore L-arginine to promote T cell proliferation and reduce suppression	Preclinical	Enhances T cell function; clinical data still limited
Imiquimod (TLR7 agonist)	PRRs/keratinocytes	Activate local innate immune response, promote antigen presentation	Approved (topical use)	Effective for superficial lesions; local only, not systemic
Synthetic biomarkers + liquid biopsy	Tumor proteases/immune signatures	Enable early detection and immune monitoring through engineered readouts	Preclinical/Emerging	Non-invasive, dynamic monitoring; still under development

In summary, recent technological and pharmacological innovations—including personalized neoantigen vaccines, innate immune modulation through PRRs, microenvironmental reprogramming, synthetic biomarkers for enhanced early detection, and AI-supported single-cell multi-omics analyses—present transformative strategies for bolstering immune surveillance in early cancer stages. Continued multidisciplinary collaboration and rigorous clinical evaluation of these emerging strategies hold significant promise for achieving effective early cancer interception and improving patient prognosis.

## 5 Future perspectives and conclusions: harnessing immune surveillance for cancer interception

The expanding understanding of immune surveillance in the early stages of cancer presents a unique opportunity to shift the paradigm from late-stage treatment to proactive immune-based prevention. Importantly, the TME acts as both a regulator and a reflection of immune surveillance status in early lesions. Immunosuppressive constituents of the TME—such as Tregs, MDSCs, and CAFs—can inhibit immune clearance and facilitate malignant transition. Future pharmacological strategies should therefore consider TME modulation as an integral component of immune-based early cancer interception. As pharmacological tools evolve, strategies such as neoantigen-based vaccines, innate immune agonists, and immune microenvironment modulators are poised to form the foundation of next-generation cancer interception. These approaches must be supported by the development of sensitive, specific biomarkers—leveraging single-cell multi-omics and artificial intelligence—to guide risk stratification, monitor immune activity, and identify optimal therapeutic windows.

Moreover, targeting specific molecular pathways such as IDO, arginase, and adenosine signaling with pharmacological inhibitors presents promising strategies for shifting the TME toward enhanced immune responsiveness. Current clinical trials involving IDO inhibitors (e.g., Epacadostat), adenosine receptor antagonists, and novel small molecules targeting arginase demonstrate initial promise. However, translating these molecular-targeted approaches to clinical practice faces significant challenges, including ensuring specificity, minimizing off-target effects, and accurately identifying early-stage biomarkers predictive of progression risk. Furthermore, ethical and clinical uncertainties remain regarding when intervention is justified, and how to balance prevention with the potential harms of premature immune activation.

Despite this promise, substantial challenges remain, including heterogeneity in precancerous lesions, incomplete knowledge of immune dynamics during early tumorigenesis, and concerns about safety and overtreatment. Addressing these issues will require coordinated efforts across disciplines, integrating insights from immunology, pharmacology, systems biology, and clinical oncology. In conclusion, pharmacologically enhancing immune surveillance at the earliest stages of tumor development offers a compelling and underexploited opportunity in cancer prevention. We argue that the future of immune-based cancer prevention lies not in reactive checkpoint blockade at advanced stages, but in subtle, stage-specific pharmacological recalibration of immune surveillance (Chen and Mellman, 2017). This approach may involve low-dose checkpoint modulation, metabolic reprogramming of tissue-resident suppressive cells, or early vaccine priming before immune exhaustion occurs. Such strategies offer a unique opportunity to preemptively stabilize immune equilibrium and delay or prevent malignant transformation. By intervening before immune escape and malignant transformation occur, we can redefine cancer care—transforming it from a reactive endeavor to a preemptive, immune-guided strategy with the potential to reduce global cancer burden and improve long-term patient outcomes. Looking forward, pharmacological modulation of immune surveillance has the potential to redefine the paradigm of cancer prevention—from generalized population screening to personalized immune-based interception. To achieve this shift, urgent gaps must be addressed, including the development of validated early-stage biomarkers, risk-adapted treatment thresholds, and long-term safety data on early immunomodulatory interventions.
